# CD36 promotes NLRP3 inflammasome activation via the mtROS pathway in renal tubular epithelial cells of diabetic kidneys

**DOI:** 10.1038/s41419-021-03813-6

**Published:** 2021-05-21

**Authors:** Yanjuan Hou, Qian Wang, Baosheng Han, Yiliang Chen, Xi Qiao, Lihua Wang

**Affiliations:** 1 Department of Nephrology, Second Hospital, Shanxi Medical University, Taiyuan, China; 2 Department of Cardiac Surgery, Shanxi Cardiovascular Hospital, Taiyuan, China; 3 Blood Research Institute, Blood Center of Wisconsin, Milwaukee, WI USA; 4 Department of Medicine, Medical College of Wisconsin, Milwaukee, WI USA

**Keywords:** Biochemistry, Cell biology

## Abstract

Tubulointerstitial inflammation plays a key role in the pathogenesis of diabetic nephropathy (DN). Interleukin-1β (IL-1β) is the key proinflammatory cytokine associated with tubulointerstitial inflammation. The NLRP3 inflammasome regulates IL-1β activation and secretion. Reactive oxygen species (ROS) represents the main mediator of NLRP3 inflammasome activation. We previously reported that CD36, a class B scavenger receptor, mediates ROS production in DN. Here, we determined whether CD36 is involved in NLRP3 inflammasome activation and explored the underlying mechanisms. We observed that high glucose induced-NLRP3 inflammasome activation mediate IL-1β secretion, caspase-1 activation, and apoptosis in HK-2 cells. In addition, the levels of CD36, NLRP3, and IL-1β expression (protein and mRNA) were all significantly increased under high glucose conditions. CD36 knockdown resulted in decreased NLRP3 activation and IL-1β secretion. CD36 knockdown or the addition of MitoTempo significantly inhibited ROS production in HK-2 cells. CD36 overexpression enhanced NLRP3 activation, which was reduced by MitoTempo. High glucose levels induced a change in the metabolism of HK-2 cells from fatty acid oxidation (FAO) to glycolysis, which promoted mitochondrial ROS (mtROS) production after 72 h. CD36 knockdown increased the level of AMP-activated protein kinase (AMPK) activity and mitochondrial FAO, which was accompanied by the inhibition of NLRP3 and IL-1β. The in vivo experimental results indicate that an inhibition of CD36 could protect diabetic db/db mice from tubulointerstitial inflammation and tubular epithelial cell apoptosis. CD36 mediates mtROS production and NLRP3 inflammasome activation in db/db mice. CD36 inhibition upregulated the level of FAO-related enzymes and AMPK activity in db/db mice. These results suggest that NLRP3 inflammasome activation is mediated by CD36 in renal tubular epithelial cells in DN, which suppresses mitochondrial FAO and stimulates mtROS production.

## Introduction

Diabetic nephropathy (DN) is one of the most severe chronic microvascular complications of diabetes mellitus, which currently represents the leading cause of end-stage renal failure worldwide^1^. Previous research suggests that tubulointerstitial inflammation plays an independent role in promoting the pathogenesis of renal function in DN; however, the precise mechanism remains unknown^2,3^. A number of studies have demonstrated that interleukin-1β (IL-1β) plays an important immune stimulatory role as a master proinflammatory cytokine in DN^3^. The NLRP3 inflammasome is an IL-1β family cytokine-activating protein complex consisting of a Nod-like receptor and ASC adapter^4^. It has been reported that the NLRP3 inflammasome can be activated by persistent high glucose (HG) conditions^5,6^. Moreover, reduced NLRP3 inflammasome levels can attenuate renal tissue inflammation associated with DN in type 2 diabetes^5,7^; however, the molecular mechanisms by which HG activates the NLRP3 inflammasome remain largely unexplored.

Multiple endogenous and exogenous stimulus signals can activate the NLRP3 inflammasome under disease conditions, the main mechanisms of which include potassium outflow, lysosomal injury, and reactive oxygen species (ROS)^8–10^. In rat models of diabetes and cultured renal cells, ROS has been shown to activate the NLRP3 inflammasome by activating thioredoxin-interacting protein^11^. The transcription factor, NF-κB, which can be activated by ROS, has also been shown to be involved in NLRP3 inflammasome activation in DN^12^. Another study found that silencing the Nrf2 gene (a transcription factor that mediates anti-oxidant genes) in podocytes increased the activation of the NLRP3 inflammasome^13^. Taken together, the literature indicates that ROS appear to play an important role in HG-mediated activation of the NLRP3 inflammasome in DN.

ROS production is often associated with an alteration in mitochondrial function and bioenergetics^14^. Renal tubular epithelial cells (TECs) are one of the most mitochondria-rich cell types, which preferentially generate ATP from fatty acid oxidation (FAO)^15^. Therefore, TECs are vulnerable to impaired mitochondrial bioenergetics^16^. Studies have shown that the expression of enzymes and transcription factors required for fatty acid metabolism in TECs is reduced in DN, and mitochondrial FAO is inhibited^16^. In addition, Chen et al.^17^ showed that decreased FAO will lead to increased production of mitochondrial ROS (mtROS), which will further drive an inflammatory status in macrophages. However, the molecular mechanisms linking mitochondrial FAO to mtROS and inflammation under diabetic conditions remain unclear. CD36 belongs to a class B scavenger receptor family, and is a glycosylated surface receptor present in the cytoplasmic membrane and mitochondria of TECs^18^. As a fatty acid transporter, CD36 mediates lipid deposition, and also participates in the inflammatory response and mitochondrial FAO, which contributes to the regulation of chronic metabolic diseases, including atherosclerosis and non-alcoholic steatohepatitis^17–20^. Our previous data indicate that CD36 can induce ROS production^21^. Whether CD36 can also promote mtROS production by regulating mitochondrial FAO in TECs remains to be established.

In this study, we used mitochondria-targeted ROS probes and ROS inhibitors to demonstrate that CD36 mediates mtROS production to activate the NLRP3 inflammasome in TECs. Mechanistically, HG-induced overexpression of CD36 inhibited FAO by inactivating AMPK, which facilitates mtROS generation.

## Materials and methods

### Cell culture and transfection

The human renal proximal tubular cell line (HK-2 cells) were obtained from the ATCC (American Type Culture Collection, Manassas, VA, USA) and maintained in Dulbecco’s modified Eagle’s medium (DMEM) containing 1 g/L glucose supplemented with 10% fetal bovine serum (FBS), 100 U/mL penicillin, and 100 mg/mL streptomycin at 37 °C in a 5% CO_2_ atmosphere. d-glucose, palmitic acid (PA), and mannitol were purchased from Sigma (St. Louis, MO, USA). HK-2 cells were fasted for 12 h and stimulated with normal glucose (NG) 5.6 mmol/L, NG plus 24.4 mM mannitol (M), and 30 mmol/L glucose (HG) for various periods of time. We investigated the metabolic changes in HK-2 cells by treating them with 30 mmol/L glucose (HG), 300 μmol/L palmitic acid (PA), and HG plus PA (HG + PA) for 72 h. HK-2 cells were transfected with NLRP3 shRNA or a control shRNA plasmid (Genechem, Shanghai, China), and treated with an FuGENE-HD transfection reagent in accordance with the manufacturer’s instructions. The expression of CD36 in HK-2 cells was knocked down or over-expressed using a lentivirus expression vector. The lentivirus constructs, including an LV3 empty vector (LV3-NC), LV3 containing CD36 (LV3-CD36), and CD36 mutant (LV3-shRNA) were provided by GenePharma (Shanghai, China). HK-2 cells were infected with a multiplicity of infection (MOI) of 10 and termed C-HK-2, hCD36-HK-2, and kCD36-HK-2, respectively.

### Experimental animals

Eight-week-old C57BLKS/J db/db diabetic (*n* = 18) and db/m (*n* = 6) normal male mice were purchased from the Model Animal Research Center of Nanjing University. The db/db mice were injected with NC lentivirus vectors (LV3-NC; *n* = 6) and CD36 mutant lentivirus vectors (LV3-shRNA; *n* = 6) in the tail vein every two weeks for a total of 8 weeks. All animals were housed in a temperature-controlled room at the animal center of Shanxi Medical University. All mice were given free access to food and water, and sacrificed at 16 weeks of age. Serum samples, 24-h urine samples, and kidney tissues were collected from each mouse for further study. All experimental protocols were conducted in accordance with the Ethics Review Committee for Animal Experimentation of Shanxi Medical University.

### Western blotting analysis

Protein was extracted from HK-2 cells or kidney tissues from the cortex using RIPA buffer, separated by SDS-PAGE, and transferred to a PVDF membrane (Millipore, Billerica, MA). The membrane was incubated with primary antibodies specific to IL-1β, casepase-1, cleaved caspase-3, AMPK, p-AMPK, ACC, p-ACC, CPT1 (1:1000 dilution, Cell Signaling Technology, Beverly, MA), CD36, NLRP3, Bcl-2, MCP-1 (1:1000 dilution, Abcam, Cambridge, UK), Bax (1:1000 dilution, Proteintech, Chicago, IL), and β-actin (1:3000 dilution, Santa Cruz, CA) overnight at 4 °C. The membranes were then incubated with goat anti-rabbit or mouse IgG horseradish peroxidase conjugate (1:10,000 dilution, Santa Cruz, CA, USA) and scanned using an Odyssey Fc System (LI-COR, USA).

### Enzyme-linked immunosorbent assay

The HK-2 cells were cultured in six-well plates under different experimental conditions, and the cell culture supernatants were collected. The IL-1β protein was quantified using a commercial Quantikine Enzyme-Linked ImmunoSorbent Assay (ELISA) kit (R&D systems Minneapolis, MN), in accordance with the manufacturer’s descriptions.

### Quantitative RT-PCR analysis

The total RNA and cDNA from HK-2 cells or kidney tissues from the cortex were prepared using TRIzol reagent (Invitrogen) and a High-Capacity cDNA Reverse Transcription Kit (Thermo Fisher Scientific) according to the manufacturer’s instructions. Quantitative real-time polymerase chain reaction (RT-PCR) analyses were performed using SYBR Premix ExTaq (Takara Bio Inc.) and Agilent Mx3000P QPCR Systems (Agilent, Palo Alto, CA, USA). Normalization was achieved using 18 s rRNA. The following primer sequences were used: human CD36: sense 5′-GCAACAAACCACACACTGGG-3′ and antisense 5′-AGTCCTACACTGCAGTCCTCA-3′; human NLRP3: sense 5′-TGAACAGCCACCTCACTT-3′ and antisense 5′-CAACCACAATCTCCGAAT-3′; human ASC: sense 5′-TGGACGCCTTGGACCTCA-3′ and

antisense 5′-GGACCTTCCCGTACAGAGCAT-3′; human caspase-1: sense 5′-ATTACAGACAAGGGTGCT-3′ and antisense 5′-GAATAACGGAGTCAATCAAA-3; human IL-1β: sense 5′-GTGGTGGTCGGAGATTCGTAG-3′ and antisense 5′-GAAATGATGGCTTATTACAGTGGC-3′; mouse cd36: sense 5-CTCCTAGTAGGCGTGGGTCT-3′ and antisense 5′-CACGGGGTCTCAACCATTCA-3′; 18s: sense 5′-ACACGGACAGGATTGACAGA-3′ and antisense 5′-GGACATCTAAGGGCATCACAG-3′.

### Terminal deoxynucleotidyl transferase-mediated dUTP nick end labeling (TUNEL) staining

Cellular apoptosis was detected using a TUNEL apoptosis detection kit (Promega corp., Madison, WI, USA) in accordance with the manufacturer’s instructions. The cells were stained with PI for nuclear counterstaining. Fluorescence images were obtained using a fluorescent microscope (Olympus BX63). For quantification of the positive TUNEL signal, a minimum of 500 HK-2 cells were counted in each well (*n* = 4), and the percentage of positively labeled cells was calculated. At least 10 regions at the corticomedullary junction in the sections from different mice of each group were determined and averaged.

### Confocal microscopy

For immunofluorescence staining, the cells were plated on cover slips, fixed with 4% formaldehyde for 15 min at 4 °C, and blocked with 5% BSA for 30 min. To permeabilize the cell membrane, the cells were incubated in 0.1% Triton X-100 for 20 min at room temperature. The cells were then incubated with the primary antibody (anti-CD36 1:50 dilution; anti-NLRP3 1:50 dilution) overnight at 4 °C. The next day, after incubating with an FITC-conjugated goat anti-rabbit secondary antibody (Molecular Probes, Invitrogen, Thermo Fisher Scientific, Waltham, MA, USA), the cell nuclei were stained with DAPI. After HG stimulation, living HK-2 cells were stained with MitoTracker Green (300 nM) for 30 min, and the cells were washed for 15 min with DMEM medium containing 10% FBS. After staining with DAPI to detect the cell nucleus, the stained cells were immediately visualized under a confocal microscope (Olympus FV 1000 Viewer).

### ROS detection

Intracellular ROS formation was detected using the fluorescence probe 2′, 7′-dichlorodihydrofluorescein diacetate (DCHF-DA, Invitrogen, Carlsbad, CA). After culturing the HK-2 cells in a six-well plate under different experimental conditions, the cells were washed, trypsinized, suspended in PBS, loaded with 10 μM DCHF-DA, and incubated at 37 °C for 30 min, followed by three washes with warm buffer. Mitochondrial ROS was detected using the specific mitochondria-targeted superoxide fluorescent probe, MitoNeoD^17,22^. Kidney tissues from the cortex was resected, trimmed, immediately chilled, homogenized in a phosphate buffered solution, and filtered to produce a single cell suspension^23^. HK-2 cells or a single cell suspension of the kidney cortex were incubated with a 5-μM MitoNeoD working solution at 37 °C for 15 min prior to flow cytometry. The measurement of intracellular and mtROS was performed using flow cytometry (BD Immunocytometry Systems, Franklin Lakes, NJ), and the analysis was performed using FlowJo software (FlowJo LLC, Oregon, USA). The median fluorescence intensity was used to estimate the average amount of the mitochondrial superoxide production. The results were obtained by comparing the percentage of cells with the mean fluorescence intensity.

### Seahorse extracellular flux assay

HK-2 cells were seeded into a specialized XF96 cell culture microplate (Seahorse Bioscience) at a density of 50,000−80,000 cells/well. The cells were exposed to different experimental conditions and the oxygen consumption rate (OCR) and extracellular acidification rate (ECAR) were measured using a Seahorse Bioscience Extracellular Flux Analyzer (Agilent). The cells were then lysed in RIPA buffer and subjected to a protein assay. The OCR and ECAR values were normalized to the protein values in each well.

### Metabolic data

Blood glucose, body weight, urine volume, albumin concentrations, and blood pressure were measured at 16 weeks of age. Urine was collected over a 24-h period, during which each mouse was placed in an individual metabolic cage. The level of urinary albumin, urinary creatinine, and serum creatinine were measured using reagent kits (BioSino Bio-technology and Science Inc., Beijing, China) in accordance with the respective manufacturer’s instructions. The 24-h urinary albumin excretion rate (UAER) = urinary albumin (μg/mL) × 24-h urine volume (mL).

### Renal pathology and immunohistochemistry

Renal tissues were fixed in 4% paraformaldehyde overnight at 4 °C, dehydrated, and embedded in paraffin. Tissue sections (2-μm-thick) were prepared for periodic acid-Schiff (PAS). The tubular area consisting of ~80 ± 100 proximal tubules in each mouse (six mice/group) were semiquantitatively measured using an ImageJ image processing and analysis system. The tubulointerstitial injury index was conducted by a pathologist in a blinded fashion, as previously described^24^. The percentage of damaged tubules (interstitial inflammation and fibrosis, tubular dilation, and cast formation) was graded from 0 to 3 as follows: 0, normal; 1, tubular lesion <25%; 2, 25−50% lesion; and 3, lesion >50%. Immunohistochemistry for CD36, MCP-1, and F4/80 on the renal sections was performed using an SP kit according to the manufacturer’s instructions, as described previously^24^. Labeling was visualized with 3,3-diaminobenzidine to produce a brown color, and the sections were counterstained with hematoxylin. Staining was analyzed under light microscopy using two independent, blinded observers. The collected images were assessed by the National Institutes of Health Image J software^25^ (http://rsb.info.nih.gov/ij/)^26^.

### Measurement of the urine malondialdehyde and 8 -hydroxydeoxyguanosine levels by an enzyme-linked immunosorbent assay

Urine specimens from each mouse were centrifuged at 1500 × *g* for 10 min to remove any particulates. The supernatants were collected and the level of urine malondialdehyde (MDA) and 8-hydroxydeoxyguanosine (8-OHdG) was detected using a commercial ELISA kit (Nanjing Jiancheng Bioengineering Institute, Nanjing, China), according to the manufacturer’s descriptions.

### Statistical analysis

Data were expressed as the mean ± SD. Differences between the groups were analyzed for statistical significance using a one-way analysis of variance (ANOVA), followed by a post-hoc test using the Tukey-Kramer method. All experiments were performed at least three times. A threshold *P*-value of <0.05 was considered significant.

## Results

### High glucose induces NLRP3-mediated IL-1β secretion, caspase-1 activation, and apoptosis

**Fig. 1:**
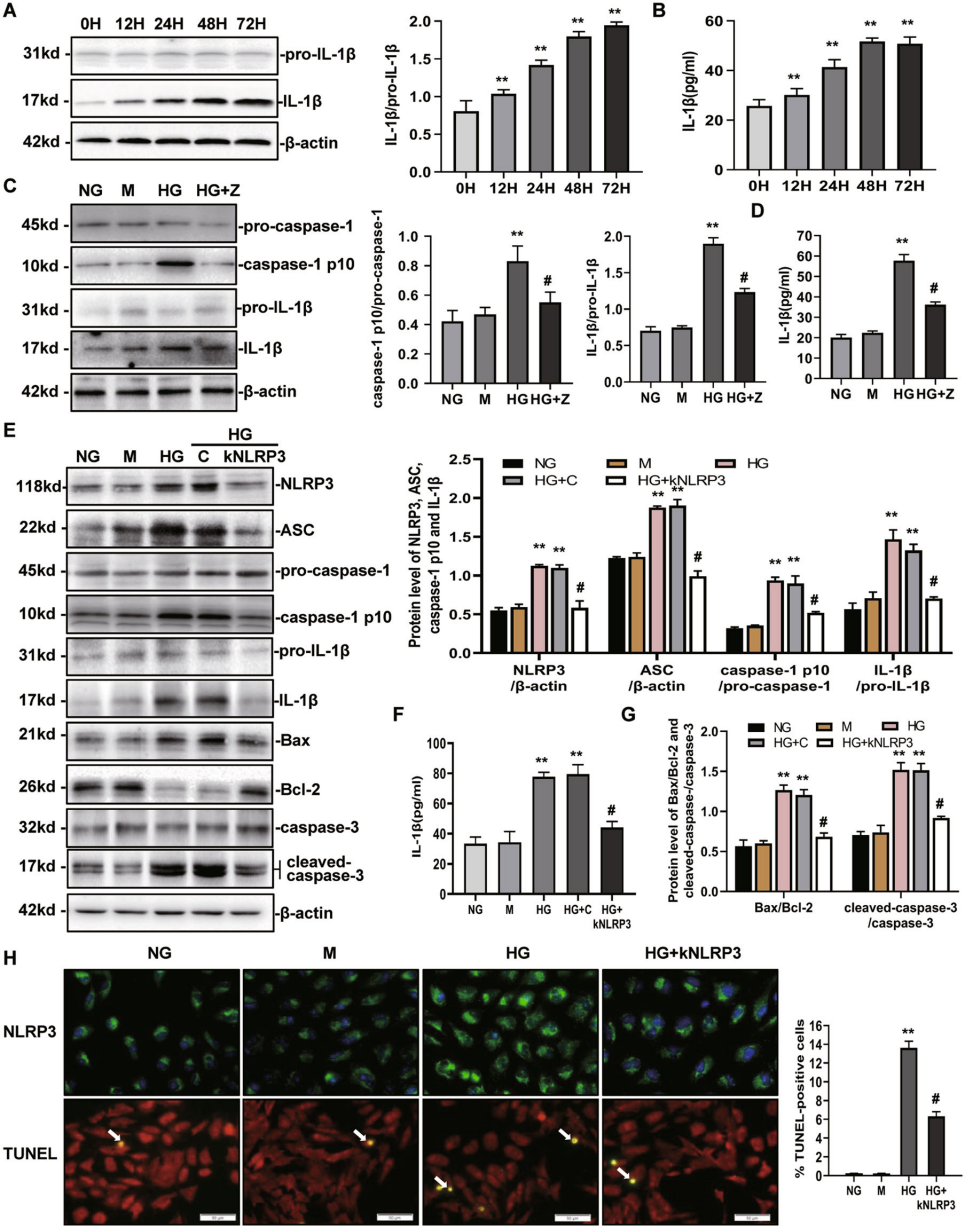
High glucose induces NLRP3-mediated IL-1β secretion and caspase-1 activation in HK-2 cells. **A**, **B** Levels of IL-1β expression in cultured HK-2 cells treated with HG (30 mM) at various time points were determined by western blot (**A**) and ELISA (**B**). **C**, **D** The protein expression of NLRP3, caspase-1, and IL-1β was analyzed by western blot (**C**) and (or) ELISA (**D**) following NG, M, HG, and HG + Z conditions for 72 h. **E−G** The levels of NLRP3, ASC, caspase-1, IL-1β, Bax, Bcl-2, and cleaved-caspase-3 protein expression were detected by western blot and/or ELISA following NG, M, HG, HG + C, and HG + kNLRP3 conditions for 72 h. **H** Immunofuorescence staining of NLRP3 and TUNEL staining in each group are shown (magnification: ×400). Bar graph indicates the mean number of TUNEL-positive cells per filed. NG: 5.6 mM d-glucose; M: NG + 24.4 mM mannitol; HG: 30 mM d-glucose; HG + Z: HG + Z-VAD-fmk (an inhibitor of pan-caspase-1; 10 μM); HG + C: HG + control shRNA plasmid; HG + kNLRP3: HG + NLRP3 shRNA plasmid. Values are expressed as the mean±SD of four independent experiments. **P < 0.01 versus the NG group; ^#^*P* < 0.05, compared with the HG group by an ANOVA.

To explore the role of NLRP3-caspase-1-IL-1β in transducing the priming signal of high glucose (HG), we first assessed the effect of HG on IL-1β induction. As shown in Fig. 1A, HG-induced IL-1β expression in a time-dependent manner. IL-1β increased significantly within 12 h and continued to rise until 72 h. IL-1β secretion in the supernatants increased following HG stimulation (Fig. 1B). Upon stimulation with HG, caspase-1 was also activated (Fig. 1C). To confirm that caspase-1 activation resulted in a marked increase in IL-1β expression in HK-2 cells, Z-VAD-fmk (pan-caspase-1 inhibitor) was used to block caspase-1 activation^27^. The level of IL-1β expression decreased significantly following the inhibition of caspase-1 (Fig. 1C, D). To confirm that NLRP3 modulated IL-1β secretion and caspase-1 activation under HG stimulation, HK-2 cells were transfected with NLRP3 shRNA to inhibit NLRP3 expression (Fig. 1E, H). In the NLRP3 shRNA-transfected HK-2 cells, HG-induced caspase-1 activation and IL-1β expression were both suppressed (Fig. 1E, F). These data suggest that the NLRP3 inflammasome-caspase-1-IL-1β axis was activated by HG in HK-2 cells. NLRP3 has been shown to mediate apoptosis signaling in contrast-induced acute kidney injury^28^ and ureteric unilateral obstruction-induced chronic kidney injury^29^. We examined whether activation of the NLRP3 inflammasome was associated with tubular epithelial cell apoptosis in vitro. As shown in Fig. 1H, the rate of TUNEL-positive HK-2 cells was increased in the HG group compared with that of the NG group; however, these alterations were significantly reversed by transfection with an NLRP3 shRNA plasmid. In addition, the level of apoptosis-associated protein expression was determined via western blot. The Bax/Bcl-2 ratio and cleaved caspase-3 was markedly increased in the HG group compared to the NG group, which was lower in the HG + NLRP3 shRNA group (Fig. 1E, G). These findings indicate that NLRP3 activation was associated with HG-elicited HK-2 cell impairment.

### High glucose levels activate NLRP3 inflammasome through CD36

**Fig. 2:**
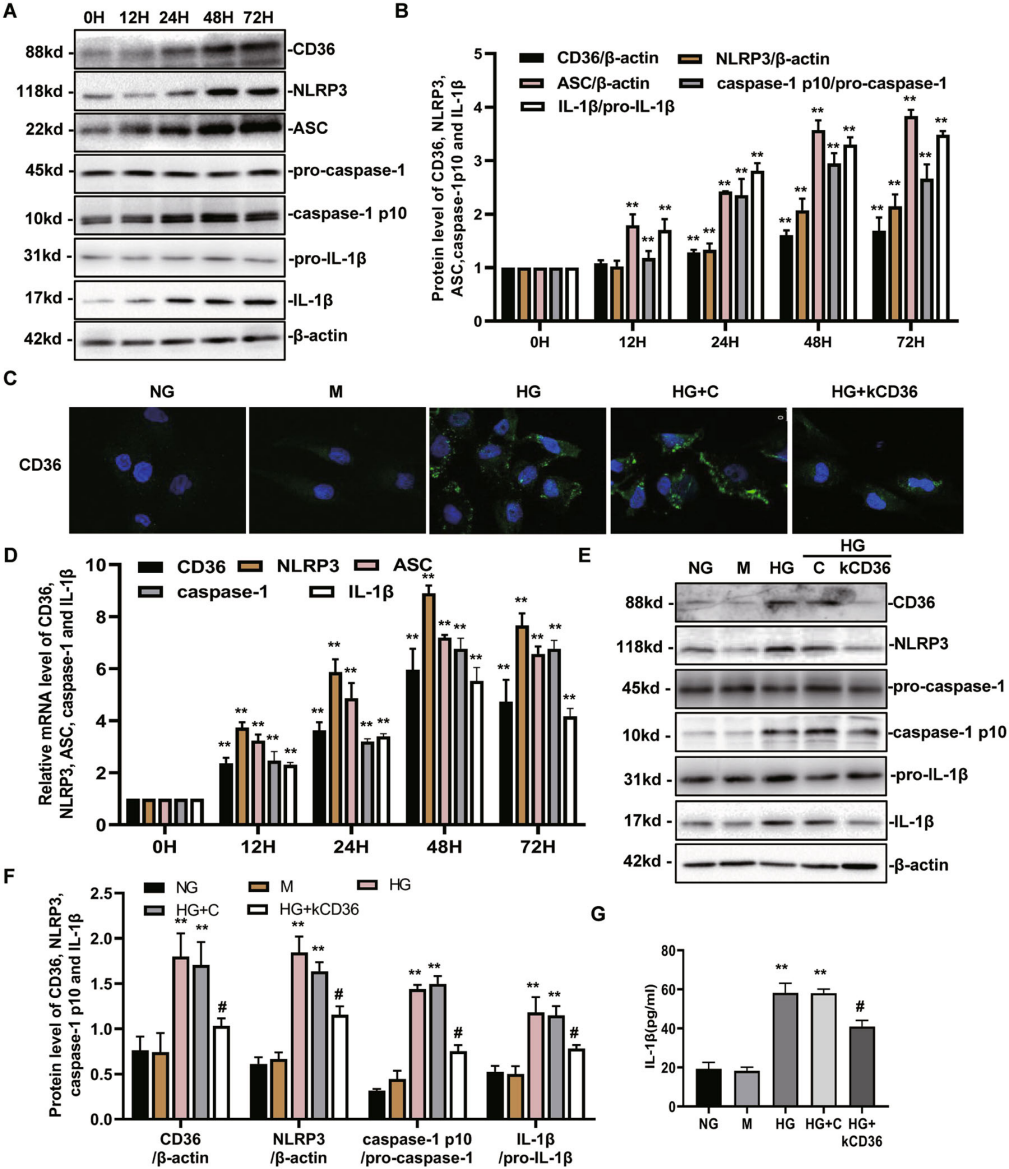
High glucose activates the NLRP3 inflammasome through CD36 in HK-2 cells. **A**, **B** The levels of CD36, NLRP3, ASC, caspase-1, and IL-1β protein expression in cultured HK-2 cells treated with HG (30 mM) at various time points were determined by western blot. **C** Immunofuorescence staining of CD36 in each group. **D** The mRNA levels of NLRP3, ASC, caspase-1, and IL-1β in cultured HK-2 cells treated with HG (30 mM) at various time points were determined by RT-PCR. **E−G** The levels of CD36, NLRP3, caspase-1, and IL-1β protein expression were detected by western blot (E) and (or) ELISA (G) under NG, M, HG, HG + C, and HG + kCD36 conditions for 72 h. NG: 5.6 mM d-glucose; M: NG + 24.4 mM mannitol; HG: 30 mM d-glucose; HG + C: HG + LV3 empty vector (LV3-NC); HG + kCD36: HG + CD36 mutant vector (LV3-shRNA). Values are expressed as the mean ± SD of four independent experiments. **P < 0.01 versus the NG group; ^#^*P* < 0.05, compared with the HG group by an ANOVA.

To further investigate the association between CD36 and NLRP3 inflammasome activation under high glucose exposure, the levels of CD36, NLRP3, ASC, caspase-1, and IL-1β mRNA and protein expression were detected by quantitative RT-PCR and western blot, respectively (Fig. 2A, B, and D). As shown in Fig. 2A, B, the level of CD36, NLRP3, ASC, caspase-1, and IL-1β protein and mRNA expression was increased in the HG groups compared with the normal group with a positive correlation. Next, lentivirus vectors (LV3-shRNA) were constructed to knockdown CD36 expression, which was validated by immunofluorescence staining (Fig. 2C). The expression of CD36 fluorescence was rarely observed in the NG group. HG stimulation for 72 h resulted in increased expression of CD36 fluorescence. In the NG group, the fluorescence was distributed in the cytoplasm of HK-2 cells; however, the fluorescence tended to be distributed around the cell membrane in the HG group. A knockdown of CD36 attenuated this fluorescence. Moreover, we also found that a knockdown of CD36 resulted in decreased NLRP3 activation and IL-1β secretion (Fig. 2E−G). These data suggest that CD36 mediates the HG-induced activation of NLRP3 inflammasomes in HK-2 cells.

### CD36 activates the NLRP3 inflammasome through mtROS

**Fig. 3:**
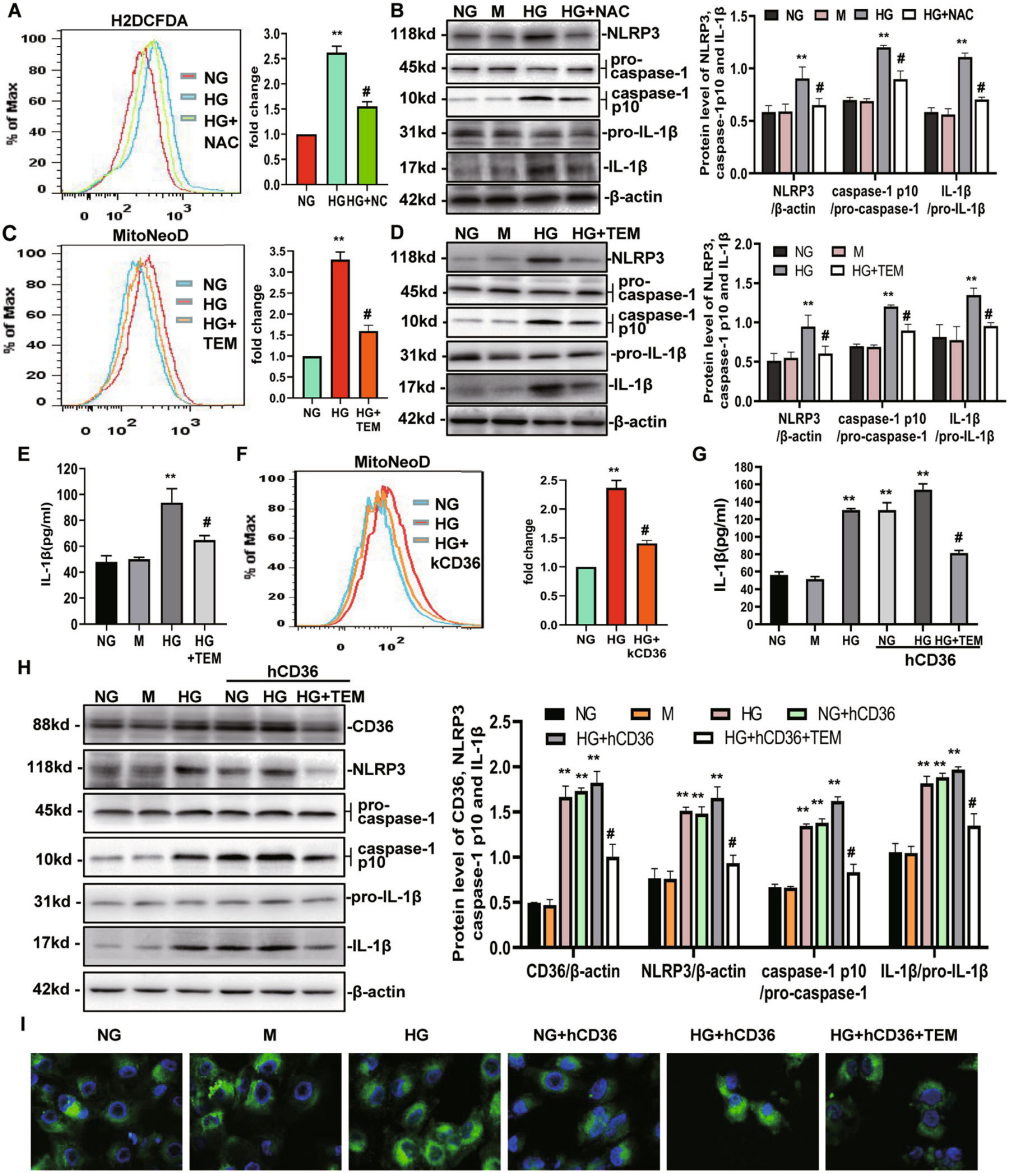
CD36 activates the NLRP3 inflammasome through mtROS. **A** The level of intracellular ROS expression in NG, HG, and HG + NAC for 72 h were detected by FACS using the DCHF-DA reagent. The MFI was quantified and shown in a bar graph. **B** Western blot for NLRP3, caspase-1, and IL-1β under NG, M, HG, and HG + NAC conditions for 72 h shown as the representative data. **C** Levels of mitochondrial ROS expression in NG, HG, and HG + TEM for 72 h were detected by FACS using the MitoNeoD reagent. **D**, **E** NLRP3, caspase-1, and IL-1β protein expression were detected by western blot (**D**) and (or) ELISA (**E**) under NG, M, HG, and HG + TEM conditions. **F** The level of mitochondrial ROS expression was detected by FACS using the MitoNeoD reagent. **G**, **H** NLRP3, caspase-1, and IL-1β protein expression were detected by western blot (H) and/or ELISA (G) under NG, M, HG, NG + hCD36, HG + hCD36, and HG + hCD36 + TEM conditions. **I** Mitochondrial images stained with Mitotracker green. NG: 5.6 mM d-glucose; M: NG + 24.4 mM mannitol; HG: 30 mM d-glucose; HG + NAC: HG + N-acetylcysteine (5 mM); HG + TEM: HG + MitoTempo (10 μM); HG + kCD36: HG + CD36 mutant vector (LV3-shRNA); NG + hCD36: NG + LV3 containing CD36 (LV3-CD36); HG + hCD36: HG + LV3 containing CD36 (LV3-CD36); HG + hCD36 + TEM: HG + hCD36 + MitoTempo (10 μM). Values are expressed as the mean ± SD of four independent experiments. **P < 0.01 versus the NG group; ^#^*P* < 0.05, compared with the HG group by ANOVA.

We next investigated whether HG-induced NLRP3 expression is ROS-dependent. ROS generation in different groups of cells was detected using a cellular ROS indicator (DCHF-DA) and the ROS signals were quantified by flow cytometry. As shown in Fig. 3A, ROS production increased following the addition of HG, which was significantly prevented by pretreatment with *N*-acetylcysteine (NAC). This finding indicated that NAC could inhibit ROS production under HG conditions. After the addition of NAC, NLRP3 inflammasome activation was detected, and the results showed that HG-induced NLRP3 activation could be inhibited by NAC (Fig. 3B). These data suggest that HG-induced NLRP3 expression is ROS-dependent. To evaluate whether mitochondrial ROS are mechanistically linked with NLRP3 inflammasome activation under HG conditions, we first tested whether mitochondria contribute to HG-induced ROS. We used a specific mitochondria-targeted superoxide fluorescent probe, MitoNeoD, and found that HK-2 cells treated with 30 mM HG exhibited increased fluorescence compared to the cells in the NG group or M group, with a ~3.4-fold increase at 72 h (Fig. 3C). The mitochondria-targeted antioxidant, MitoTempo, which is a superoxide dismutase mimetic that accumulates in mitochondria, reduced ~90% of the increased fluorescence at 72 h (Fig. 3C), and also decreased NLRP3 activation and IL-1β secretion in HK-2 cells (Fig. 3D, E).

We have previously shown that CD36 can induce intracellular ROS production in HG-induced HK-2 cells^21^. To demonstrate the role of mitochondria in this pathway, a CD36 mutant (LV3-shRNA) lentivirus vector was transfected into HK-2 cells, followed by an incubation with HG for 72 h, and the level of mtROS was detected using the specific mitochondria-targeted superoxide fluorescent probe, MitoNeoD. As shown in Fig. 3F, the CD36 knockdown significantly inhibited HG-induced mtROS by ~50% at 72 h. To further test whether CD36 activates the NLRP3 inflammasome through mtROS, we used a lentivirus vector to overexpress CD36 (LV3-CD36), which upregulated the level of CD36 protein expression by approximately 3~4 fold in HK-2 cells prior to HG stimulation (Fig. 3H). We found that the activation of NLRP3 was increased (Fig. 3H) in conjunction with IL-1β secretion (Fig. 3G), which were blocked by MitoTempo (Fig. 3H and G). The results of mitochondrial staining by MitoTracker Green showed that there were no significant differences in the level of the mitochondrial content among the groups (Fig. 3I). These data support a critical role of mtROS for CD36-induced inflammasome activation in HK-2 cells.

### HG induces a metabolic switch from mitochondrial oxidative phosphorylation to glycolysis, which facilitates mtROS production in HK-2 cells

**Fig. 4:**
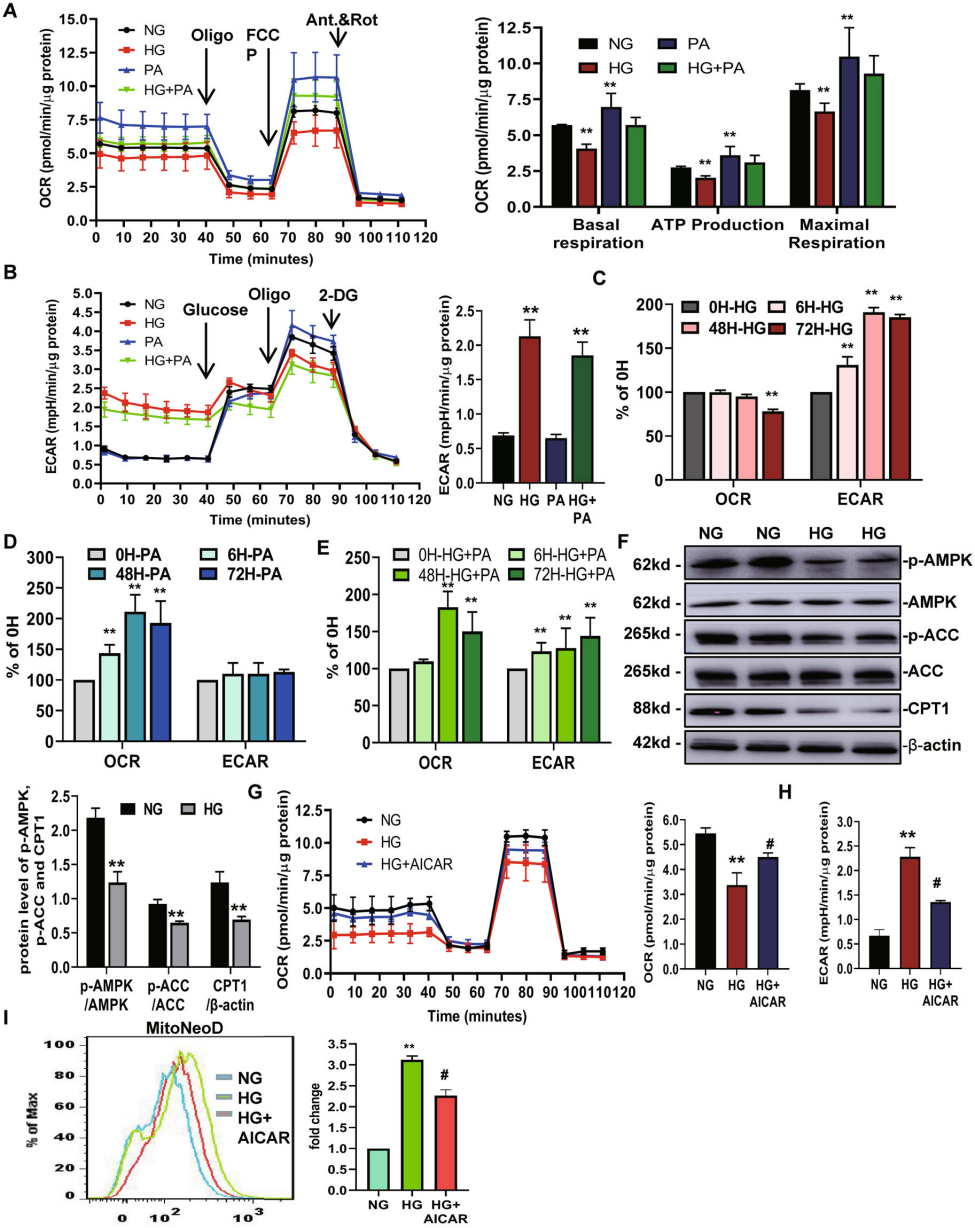
High glucose induces a metabolic switch from mitochondrial oxidative phosphorylation (OXPHOS) to glycolysis, which facilitates mtROS production in HK-2 cells. **A**−**E** The metabolic status of the HK-2 cells was determined by evaluating the oxygen consumption rates (OCR) (**A**, **C**–**E**), and the extracellular acidification rate of the media (ECAR) (**B**–**D** and **E**) using Agilent Seahorse XF technology. **A**, **B** The left panels show the representative OCR (**A**) and ECAR (**B**) curves from HK-2 cells treated with NG, HG, PA, and HG + PA for 72 h. Right panels show the quantified data from replicate studies (*n* = 4 per group). **C**−**E** The levels of OCR and ECAR were quantified from HK-2 cells treated with HG, PA, HG + PA at various time points (*n* = 4 per group). **F** Levels of p-AMPK, p-ACC, and CPT1 expression from HK-2 cells treated with culture medium or 30 mM d-glucose for 72 h. **G**, **H** OCR curve and OCR, ECAR quantified data from HK-2 cells under NG, HG, and HG + AICAR conditions. **I** The level of mtROS expression was detected by FACS using MitoNeoD reagent. NG: 5.6 mM d-glucose; HG: 30 mM d-glucose; PA: NG + 300 μM palmitic acid; HG + PA: HG + 300 μM palmitic acid; HG + AICAR: HG + AICAR (AMPK activator; 1 mM). Values are expressed as the mean ± SD. **P < 0.01 versus the NG group by an ANOVA.

Mitochondrial ROS production is often associated with bioenergetic alterations. Thus, we next investigated the metabolic changes in HK-2 cells by treating them with 30 mM glucose, 300 μM palmitic acid (PA), and HG plus PA for 72 h, followed by a Seahorse extracellular flux analysis, which the measures oxygen consumption rate (OCR) and extracellular acidification rate (ECAR). We found that the OCR was markedly higher after the PA was added to the HK-2 cells for 72 h, indicating that HK-2 cells efficiently metabolize PA (Fig. 4A). HG suppressed the OCR for 72 h, with ~28% suppression. The level of basal respiration, ATP production, and maximal respiration were significantly decreased following HG treatment (Fig. 4A). These results revealed an inhibition of mitochondrial oxidative phosphorylation (OXPHOS). Concomitant with the suppression of OCR, HG stimulation for 72 h induced an elevation in ECAR, which was consistent with a metabolic switch to glycolysis (Fig. 4B). Even when simultaneously exposed to HG and PA, the cells still preferred to use glucose as an energy source, which was supported by high levels of ECAR observed in the HK-2 cells in HG + PA group (Fig. 4B). The time course experiments showed that the HG-induced increase in ECAR was apparent at 6 h post-HG exposure, whereas a decrease in the OCR was apparent at 72 h, which lagged behind (Fig. 4C).

To further study the causes of metabolic reprogramming induced by HG, we tested the protein levels of key FAO regulators in HG-induced HK-2 cells. The level of CPT1 expression and ACC phosphorylation were markedly lower in HG-induced HK-2 cells. Western blot results also confirmed a lower level of AMPK phosphorylation, which was a key regulator in the maintenance of cellular fatty acid homeostasis and controls fatty acid β-oxidation in mitochondria (Fig. 4F).

We next sought to further examine whether a decrease in mitochondrial FAO can promote metabolic changes in renal tubular cells induced by HG, which can promote increased mtROS production. We used 5-aminoimidazole-4-carboxamide-1 riboside (AICAR; an AMPK activator) to increase FAO. The Seahorse extracellular flux analysis showed that pharmacological normalization of HG-induced FAO repression with AICAR could significantly attenuate the reduced OCR induced by HG (Fig. 4G), consistent with an amelioration of mitochondrial OXPHOS. AICAR was also associated with a significant decrease in the level of HG-induced ECAR, consistent with inhibition of the metabolic switch to glycolysis (Fig. 4H). Next, we aimed to determine the role of decreased mitochondrial FAO on promoting metabolic changes, which promotes increased mtROS production. The level of mtROS detected using MitoNeoD was increased in the HG group compared with the NG group; however, it was also suppressed by AICAR (Fig. 4I).

### Inhibition of CD36 enhances mitochondrial fatty acid oxidation and ameliorates the metabolism reprogramming induced by HG

**Fig. 5:**
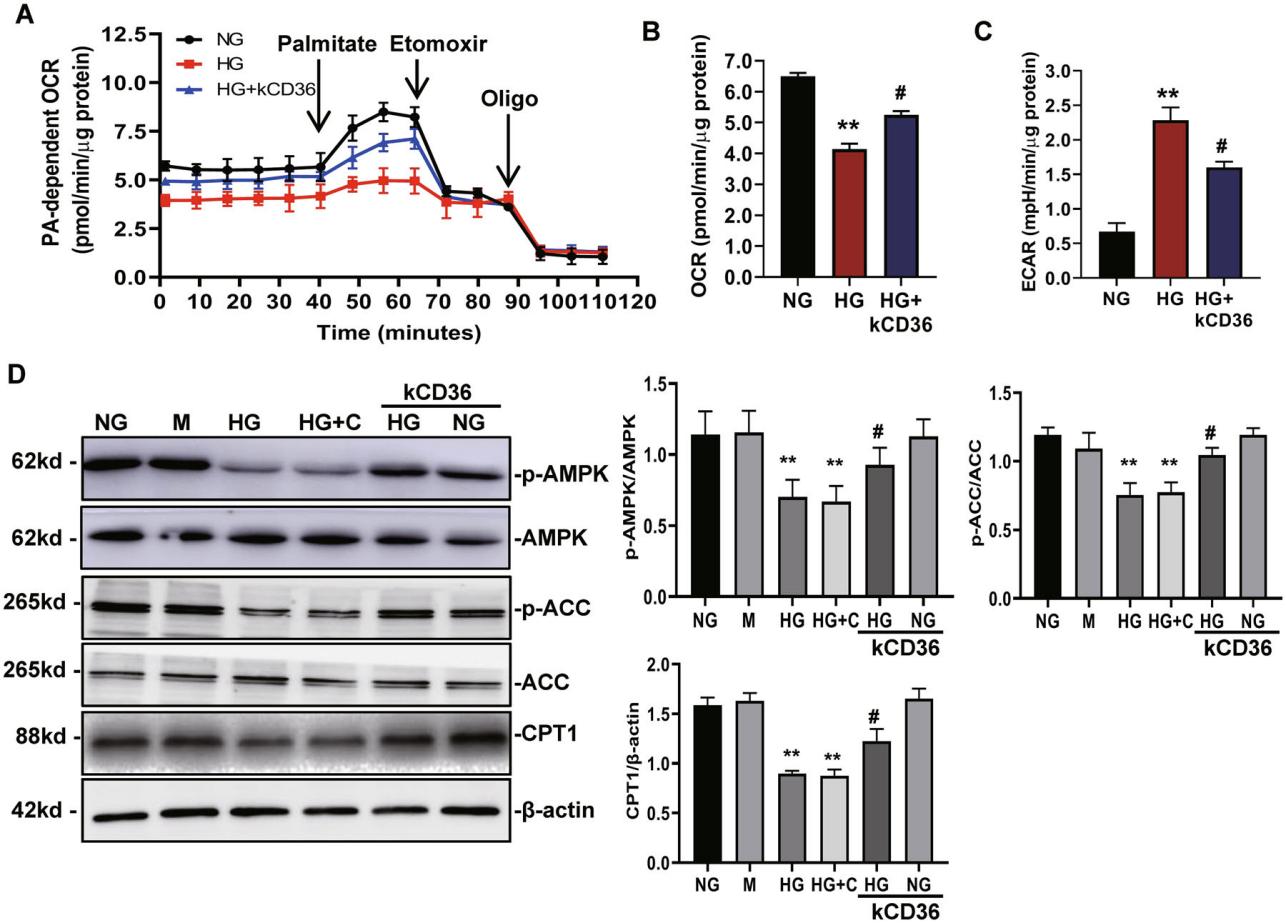
Inhibition of CD36 enhances mitochondrial fatty acid oxidation and ameliorates the metabolism reprogramming induced by HG. **A−C** FA-driven oxygen consumption rate (PA-dependent OCR) curve (**A**) and OCR, ECAR (**B**, **C**) quantified data from HK-2 cells under NG, HG, and HG + kCD36 conditions. **D** The levels of p-AMPK, p-ACC, and CPT1 were analyzed by western blot. NG: 5.6 mM d-glucose; M: NG + 24.4 mM mannitol; HG: 30 mM d-glucose; HG + C: HG + LV3 empty vector (LV3-NC); HG + kCD36: HG + CD36 mutant vector (LV3-shRNA); NG + kCD36: NG + CD36 mutant vector (LV3-shRNA). Data are expressed as the mean ± SD of four independent experiments. **P < 0.01 versus the NG group; ^#^*P* < 0.05, compared with the HG group by ANOVA.

Next, we measured the FA-driven oxygen consumption rate (PA-dependent OCR) to assess the effects of CD36 on FAO. As shown in Fig. 5A, a knockdown of CD36 in HK-2 cells was consistently associated with higher PA-dependent oxygen consumption under HG conditions compared to HG-induced HK-2 cells, which was correlated with increased FAO. The results of the Seahorse extracellular flux analysis showed a marked increase in the OCR (Fig. 5B) and decrease in ECAR (Fig. 5C) in CD36-knockdown HG-induced HK-2 cells, which was consistent with the protection of mitochondrial health. A knockdown of CD36 can make cells rely on FAO to a greater extent under a HG environment. We examined the FAO-related enzymes and pathways in HG-induced HK-2 cells. In the kCD36-HK-2 group, p-ACC and CPT1 were increased compared with the HG-HK-2 cells (Fig. 5D). The level of AMPK phosphorylation was higher in the kCD36-HK-2 cells compared to that in the HG-HK-2 cells (Fig. 5D), suggesting that an inhibition in CD36 results in activation of the AMPK pathway.

### CD36 knockdown prevents renal tubular injury, tubulointerstitial inflammation, and oxidative stress in diabetic mice

**Fig. 6:**
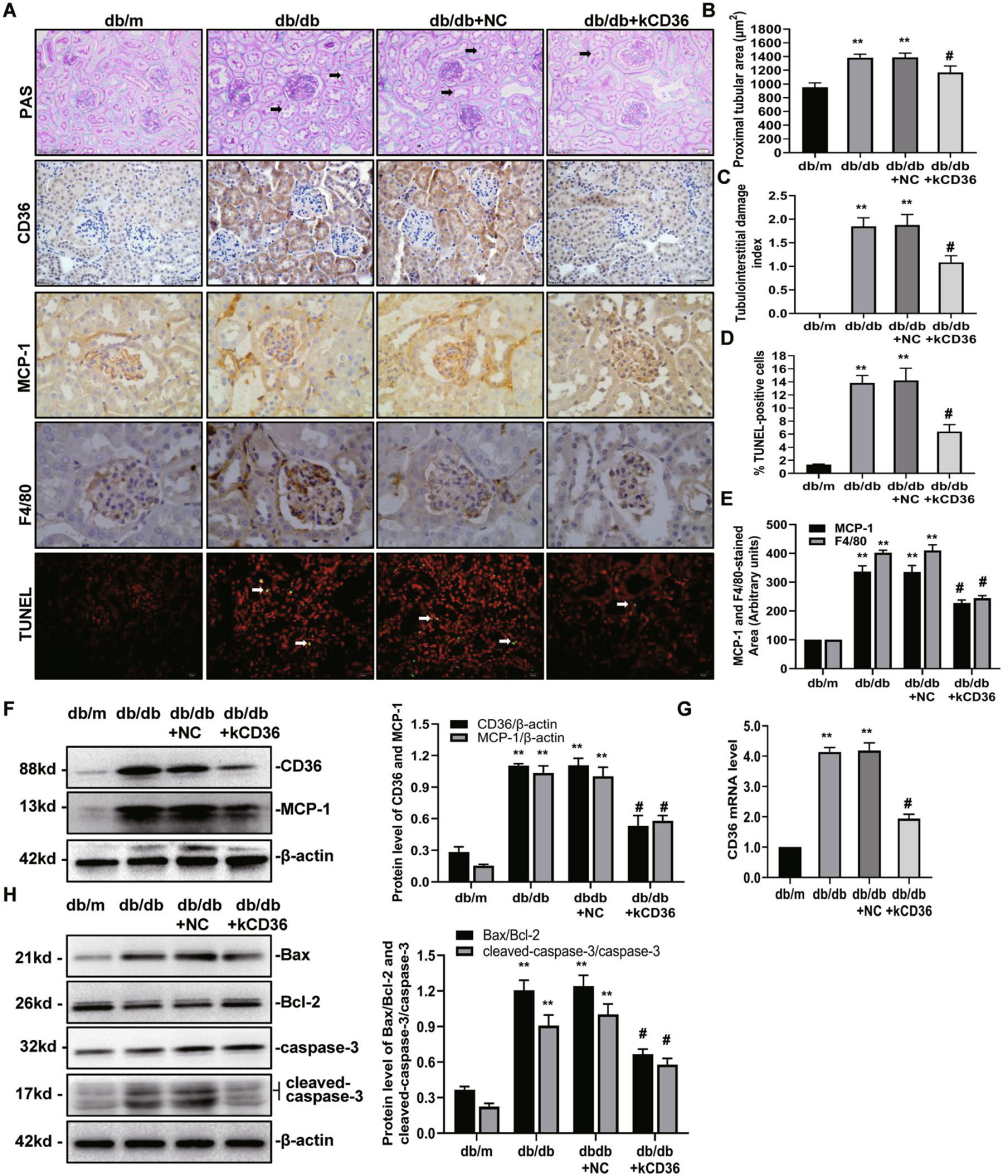
CD36 knockdown prevents tubulointerstitial inflammation in diabetic mice. **A** Representative photomicrographs of PAS staining, immunohistochemistry and TUNEL staining images in the db/m, db/db, db/db + NC, and db/db + kCD36 groups at 16 weeks (magnification: ×400). **B** Semiquantitative analyses of the proximal tubular area according to the outer diameter at 16 weeks. **C** Semiquantitative analyses of the tubulointerstitial damage index at 16 weeks. **D** Bar graph of the mean number of TUNEL-positive cells per group. **E** Semiquantitative analysis of MCP-1 and F4/80 from the immunohistochemical staining data (8–10 sections per mouse were analyzed) at 16 weeks. **F** Representative Western blot of CD36 and MCP-1 expression in the kidney. **G** The level of CD36 mRNA in the kidney tissue by RT-PCR. **H** Representative western blot of Bax, Bcl-2, and cleaved-caspase-3 expression in the kidney. db/m: normal male mice; db/db: diabetic mice; db/db + NC: db/db + NC lentivirus vectors (LV3-NC); db/db + kCD36: db/db + CD36 mutant lentivirus vectors (LV3-shRNA). Data are expressed as the means ± SD. (*n* = 6). **P < 0.01 versus the db/m group; ^#^*P* < 0.05, compared with the db/db group by ANOVA.

To further investigate the role of CD36 and its function regarding activation of the inflammasome in vivo, we injected db/db mice with lentivirus vectors (LV3-shRNA) to knockdown CD36 expression, which was validated in HK-2 cells. As shown in Fig. 6A, the expression of CD36 in the kidney was significantly increased in the diabetic db/db mice compared with the non-diabetic db/m group. CD36 expression was significantly decreased in the diabetic CD36-knockdown (db/db + kCD36) mice compared with db/db mice. The level of CD36 protein and mRNA expression were all significantly inhibited by infection with CD36 mutant (LV3-shRNA) lentivirus in db/db mice (Fig. 6F, G).

The general characteristics of the mice at the end of the experiment are shown in Table 1, the blood glucose and kidney/body weight ratio were significantly elevated in all db/db mice compared with the non-diabetic db/m control mice, but were reduced in the diabetic CD36-knockdown mice. Biochemical parameters (e.g., 24-h urine albumin excretion [UAE], urine albumin/creatinine ratio [UACR], and serum creatinine) were all significantly higher in the db/db mice compared with those in the db/m group. These increases were rescued by a CD36 knockdown. In addition, the systolic blood pressure (BP) was similar in all of the groups.

**Table 1:** Change of basic parameters in each group.

Group (μg·mg^−1^)	BG (mmol·L^−1^)	BP (mmHg)	KW/BW (mg/g)	Scr (μmol·L^−1^)	UAE (μg·24 h^−1^)	UACR
db/m	6.32 ± 0.42	121.28 ± 14.23	3.21 ± 0.98	21.49 ± 2.45	5.32 ± 1.12	12.43 ± 2.16
db/db	21.85 ± 4.51^*^	117.98 ± 13.57	5.41 ± 1.03^*^	48.41 ± 8.01^*^	301.65 ± 14.23^*^	117.24 ± 14.32^*^
db/db + NC	20.91 ± 3.82^*^	120.32 ± 12.52	6.01 ± 1.21^*^	51.21 ± 9.07^*^	297.89 ± 12.53^*^	120.78 ± 9.32^*^
db/db + kCD36	14.23 ± 2.43^#^	123.52 ± 10.89	4.53 ± 0.84^#^	32.42 ± 2.54^#^	211.46 ± 36.23^#^	75.68 ± 9.87^#^

Date are presented as the mean ± SEM (*n* = 6).

*BG* blood glucose, *BP* blood pressure, *KW/BW* kidney weight/body weight, *Scr* serum creatinine, *UAE* urine albumin excretion, *UACR* urine albumin/creatinine ratio.

*P < 0.05 versus db/m; ^;#^*P* < 0.05 versus db/db.

To investigate the role of CD36 in the progression of renal tubular injury, PAS staining was used to assess the tubular injury scores in each group. We found that the proximal tubular area became larger in the db/db mice compared to that of the db/m mice. The diabetic CD36-knockdown mice exhibited reduced tubular injury compared to the diabetic db/db mice (Fig. 6A, B). Tubulointerstitial damage was increased compared with the db/m control mice, and a knockdown of CD36 attenuated the tubular damage in the kidneys of the db/db mice (Fig. 6A, C). TUNEL-positive tubular epithelial cells were significantly increased in the kidneys of db/db mice compared with the normal control db/m mice, whereas the rate was significantly reduced in the CD36-knockdown mice (Fig. 6A, D). The level of apoptosis-associated Bax/Bcl-2 ratio and cleaved caspase-3 were markedly increased in db/db mice group compared to the db/m group, which was also reduced in CD36-knockdown mice (Fig. 6H).These findings indicate that CD36 expression is associated with the apoptotic events of proximal tubular cells, suggesting that CD36 may play a role in renal tubular injury in diabetic kidneys.

To explore whether CD36 is involved in renal inflammation in the diabetic state in vivo, we observed the level of MCP-1 and F4/80 expression in the kidneys of each group. The expression of MCP-1 was significantly elevated in the diabetic db/db mice compared with the non-diabetic db/m mice. This effect could be markedly ameliorated by a knockdown in CD36 (Fig. 6A, E, and F). F4/80 is a macrophage marker, and the number of infiltrating F4/80-positive macrophages reflects the degree of tubulointerstitial inflammation. We found that infiltration of diabetic-induced F4/80-positive cells was ameliorated in the kidneys of CD36-knockdown mice (Fig. 6A, E).

**Fig. 7:**
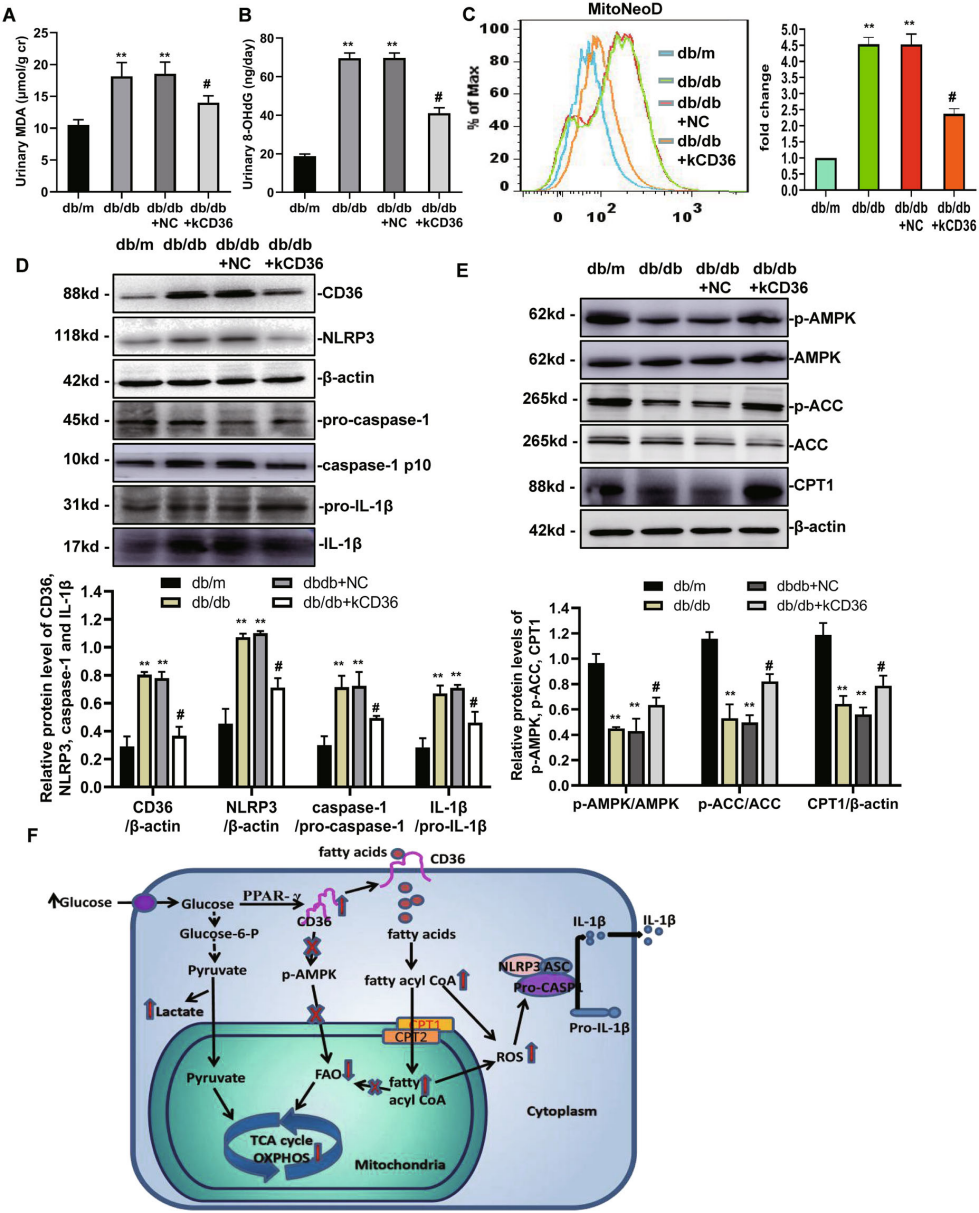
CD36 knockdown inhibits diabetes-induced oxidative stress, NLRP3 inflammasome formation, phosphorylation of AMPK, ACC, and expression of CPT1 in db/db mice. **A** The level of urinary MDA was detected by ELISA. **B** The level of urinary 8-OHdG was detected by ELISA. **C** Level of mtROS expression in experimental animals was determined by FACS using MitoNeoD reagent. **D** Representative western blot and quantitative analysis of the level of CD36, NLRP3, caspase-1, and IL-1β expression in the kidney. **E** The level of p-AMPK, AMPK, p-ACC, ACC, and CPT1 protein expression was analyzed by western blot. db/m: normal male mice; db/db: diabetic mice; db/db + NC: db/db + NC lentivirus vectors (LV3-NC); db/db + kCD36: db/db + CD36 mutant lentivirus vectors (LV3-shRNA). Data are expressed as the means ± SD (*n* = 6). **P < 0.01 versus the db/m group; ^#^*P* < 0.05, compared with the db/db group by ANOVA. **F** Molecular mechanism by which CD36 stimulates activation of the NLRP3 inflammasome in renal TECs in DN. CD36 expression was enhanced, which occurred in the presence of high glucose. Inhibition of FAO through inactivation of the AMPK signaling pathway promotes decreased OXPHOS. Under HG conditions, the CD36-induced decrease in FAO facilitates increased glycolysis, an imbalance between FA uptake and consumption (FAO), and contributes to mtROS production, which activates the NLRP3 inflammasome signaling pathway.

To test the relevance of CD36 and diabetes-induced oxidative stress in vivo, we measured the level of urinary MDA and 8-OHdG by an ELISA. The results showed a significant increase in the level of MDA and 8-OHdG for the db/db group compared with the control db/m group, which could be reduced by a knockdown of CD36 (Fig. 7A, B). To test whether CD36 was linked with mtROS in TECs in vivo, we determined the changes in mtROS levels in the kidney cortex (~90% proximal tubules) of mice in each group using MitoNeoD, respectively. Accordingly, we found that there was a significant increase in green fluorescent signals in the kidney of db/db mice compared with db/m mice. However, these levels were not as high in the CD36-knockdown mice, which was consistent with our in vitro findings (Fig. 7C).

### CD36 knockdown inhibits renal NLRP3 inflammasome formation, upregulates the phosphorylation of AMPK, ACC, and expression of CPT1 in db/db mice

Our in vitro experiments demonstrated that activation of the NLRP3-ASC-caspase-1-IL-β axis plays an important role in HK-2 cells following HG stimulation. Therefore, we evaluated the role of CD36 in NLRP3 inflammasome activation in diabetic kidneys. As shown in Fig. 7D, the protein level of NLRP3, caspase-1, and IL-1β expression in the db/db mice kidneys were increased compared with db/m mice, which were significantly inhibited by a CD36 knockdown.

To elucidate the mechanisms by which CD36 regulates FA metabolism in vivo, we examined FAO-related enzymes and associated pathways in diabetic kidneys. The level of renal expression of p-AMPK, p-ACC, and CPT1 were significantly downregulated in diabetic db/db mice compared with the non-diabetic db/m group, and a knockdown of CD36 significantly increased their levels (Fig. 7E). These findings were also consistent with our in vitro findings.

## Discussion

In the present study, we reported both ex vivo and in vivo data showing that CD36 stimulates NLRP3 inflammasome activation through mtROS in renal tubular epithelial cells in DN. We also found that HG induces a metabolic switch from OXPHOS to glycolysis in renal tubular cells, which facilitates mtROS production. We further demonstrate that CD36 inhibits the mitochondrial FAO mediated by this metabolic reprogramming, thereby promoting mtROS production. Our study explored the novel mechanism of HG-mediated activation of NLRP3 inflammasomes, as well as the use of CD36 as a potential therapeutic target for DN.

In general, it has been recognized that tubulointerstitial inflammation is the characteristic pathological alteration downstream of DN pathogenesis, which often results in the development of renal interstitial fibrosis and a loss of renal function^2,3^. The effects of NLRP3 inflammasome activation in DN has been the subject of investigation in recent years^4,7^. NLRP3 inflammasome activation and secretion of IL-1β can promote the development of tubular interstitial inflammatory responses^30^. Recent studies have shown that proteinuria causes NLRP3 inflammasome activation and IL-1/IL-18 maturation in a time course- and dose-dependent manner in the proximal tubules^31^. Further investigation has indicated that Ang II can also induce NLRP3 inflammasome activation in TECs, which is associated with mitochondrial dysfunction^32^. Moreover, Chen et al.^33^ found that NLRP3 inflammasome in renal TECs is activated by the purine receptor, P2X4, and regulates IL-1β secretion and release under HG stimulation. In this study, we found that IL-1β expression and secretion were increased in HG-induced HK-2 cells in a time-dependent manner. The inhibition of the NLRP3 inflammasome and caspase-1 further proves that the NLRP3 inflammasome-caspase-1-IL-1β axis plays a role in HK-2 cell injury stimulated with HG, and plays an important inflammatory role in the development of DN.

CD36 is a transmembrane glycoprotein molecule that plays an important role in the transmembrane transport of long-chain fatty acids in the body and the occurrence of metabolic inflammation^18^. Sheedy et al. reported that in the pathogenesis of atherosclerosis, Alzheimer’s disease, diabetes, and other aseptic inflammation-related diseases, CD36 participates in activation of the NLRP3 inflammasome by recognizing ox-LDL, amyloid-β, and pancreatic amyloid proteins^34^. Subsequent studies have also confirmed the presence of CD36 as a key regulatory molecule upstream of the NLRP3 inflammasome in ox-LDL-induced retinal endothelial cells and fructose-induced cardiomyocytes in the inflammatory response^35,36^. Our studies extend the scope of activation of NLRP3 inflammation mediated via CD36 in renal TECs. ROS are well-known activators of the NLRP3 inflammasome^10^, particularly in DN^4,37^. Our results showed that NAC could inhibit ROS production in HG-induced HK-2 cells. NAC blocks the activation of NLRP3 and IL-1β secretion by scavenging cellular ROS. A recent study reported that mtROS overproduction is associated with an increase in NLRP3/IL-1β expression in the kidneys of patients with DN^38^. Our findings in renal TECs are in agreement with those which have reported that the mtROS scavenger, MitoTempo, can significantly inhibit HG-induced activation of the NLRP3 inflammasome. Therefore, CD36 appears to play a role as an activator of NLRP3, whereas mtROS plays a major role. In addition, activation of the NLRP3 inflammasome occurs in two steps. In the first step, PAMPs are recognized by pathogen recognition receptors (PRRs), which subsequently activate NF-κB, leading to increased levels of NLRP3 activation and IL-1β secretion. In the second step, several stimuli can induce the formation of the NLRP3 inflammasome and its subsequent activation to produce mature IL-1β. Our research shows that mtROS inhibition by MitoTempo could reduce the level of NLRP3 protein and mature IL-1β production, whereas the level of NLRP3 mRNA and pro-IL-1β did not substantially change (data not shown). Although the mechanism by which ROS activates the NLRP3 inflammasome requires further research, mtROS may be critica for the second step of NLRP3 inflammasome activation.

The production of mtROS is closely related to mitochondrial metabolism and functionality, especially in renal TECs^39^. Tubular cells have a high energy demand and possess a large number of mitochondria^40^. In addition, ATP used by TECs is primarily produced by mitochondrial FAO. In this study, We examined the metabolic changes that occurred in HK-2 cells under environments with different energy supply requirements. We also found that renal TECs are critically dependent on FAO as their energy source, which was in line with a Seahorse extracellular flux analysis showing that HK-2 cells can preferentially take up PA to produce ATP. HG induces a metabolic switch from mitochondrial OXPHOS to glycolysis. Despite having sufficient lipids, the cells continue to maintain this metabolic switch. Increased glucose utilization is the key metabolic phenotype of polycystic kidney disease and renal cell cancer^41,42^. However, renal TECs did not markedly revert to glucose oxidation in CKD, and the signaling mechanisms lose the capacity for FAO^16^. The key observation in the current study is that decreased FAO is also the main mechanism of an HG-induced metabolic switch, which contributes to increased mtROS.

We also found that CD36 appears to be an upstream modulator of FAO in renal TECs. CD36 inhibited the levels of FAO in HK-2 cells, which facilitates a metabolic switch, and aggravates mtROS production, promoting the activation of subsequent NLRP3 inflammasomes and secretion of inflammatory factors. A similar mechanism has already been described in oxLDL-induced macrophages: CD36 drives the dysregulation of FA metabolism to oxidative stress from the mitochondria, which drives NF-κB activation and the generation of inflammatory cytokines^17^. Current studies on the mechanism of CD36 in renal tubular cells have mainly focused on lipid toxicity induced by fatty acid uptake and transport^43–45^; however, increasing evidence has shown that CD36 can regulate the oxidation of mitochondrial fatty acids and maintain the balance of the cell metabolism^46–49^. For example, in skeletal and cardiac cells, CD36 regulates AMPK signaling through the LKB1 pathway to regulate the oxidative metabolism of fatty acids^46^. Our previous study also found that SS31 (a mitochondrial protective agent) can alleviate the renal inflammatory response by inhibiting CD36 expression^24^. A recent study has demonstrated that in non-alcoholic fatty liver disease, inhibition of CD36 palmitoyl modification increases mitochondrial FAO by activating AMPK, which reduces inflammation in the liver tissue^20,49^. In this study, our results showed that CD36 loss can significantly upregulate AMPK phosphorylation and may be a modulator of FAO. AMPK is a key protein that regulates fatty acid oxidative metabolism. It is generally believed that AMPK activation can inhibit ACC activity and increase the fatty acid oxidative metabolism by reducing the inhibitory effect of malonyl coenzyme A on CPT-1^50,51^; however, the precise mechanism by which CD36 regulates FAO in renal TECs requires further study.

Based on the present findings, we postulate that enhanced CD36 expression, which occurred in the presence of HG, inhibited FAO through inactivation of the AMPK signaling pathway promotes decreased OXPHOS. The CD36-induced decrease in FAO facilitates increased glycolysis and an imbalance between FA uptake and consumption (FAO). This decrease in FAO contributes to the production of mtROS, which can activate the NLRP3 inflammasome signaling pathway (Fig. 7H). In summary, the results of the present study provides evidence suggesting that HG-induced NLRP3 inflammasome activation is mediated by CD36, which suppresses mitochondrial FAO, and stimulates mtROS production. CD36 is a potential therapeutic target for DN.
